# Severe Decompression Illness: Case Report, Prehospital Recognition, and Regional Transport Considerations

**DOI:** 10.1155/2017/7203085

**Published:** 2017-10-04

**Authors:** Julie Estrada, David Meurer, Kevin De Boer, Karl Huesgen

**Affiliations:** ^1^Emergency Medicine Residency Program, Department of Emergency Medicine, University of Florida, Gainesville, FL, USA; ^2^Department of Emergency Medicine, Division of Emergency Medical Services, University of Florida, Gainesville, FL, USA; ^3^ShandsCair Critical Care Transport Program, UF Health, Gainesville, FL, USA; ^4^Florida Hospital/Central Florida Pulmonary Group, Orlando, FL, USA

## Abstract

A 46-year-old male presented to our tertiary care emergency department (ED) with shortness of breath and chest pain following an uneventful four-hour SCUBA dive at 100 feet. His prehospital emergency medical services (EMS) assessment revealed transient hypotension and hypoxia. He later developed progressive skin mottling. Serology was significant for acute kidney injury, transaminitis, hemoconcentration, and hypoxia on an arterial blood gas. Computed tomography (CT) angiography demonstrated intravascular gas throughout the mesenteric and pulmonary arteries as well as the portal venous system. No abnormality was seen on head CT and the patient had normal mental status. Prehospital nonrebreather oxygen therapy was changed to continuous positive airway pressure (CPAP) upon ED arrival, and the patient was intubated prior to transfer to a hyperbaric facility. However, within 24 hours the patient was found to have multiorgan failure, diffuse cerebral edema, and brain death despite no further episodes of hypotension or hypoxia. No intracranial gas was seen on repeat head CT. Our case demonstrates the importance of early recognition of decompression illness by EMS personnel, consideration of ground versus flight transportation of these patients to the nearest hyperbaric center, and the possible use of prehospital CPAP as an alternative to enhance oxygenation.

## 1. Introduction

Decompression illness is caused by the release of dissolved gas from blood following underwater diving causing clinical manifestations. It is a function of Henry's Law in which a pressure reduction while ascending after a dive can cause dissolved nitrogen to be released into the tissues and blood, causing intravascular and extravascular bubble formation [1]. It is uncommon if the diver has followed the appropriate procedures during ascent. The effects of these bubbles typically range from mild pain to complete vascular obstruction, with possible interference in hemodynamics and respiration. Secondary effects may be due to endothelial damage with resultant capillary leakage, hemoconcentration, neutrophil activation, and platelet activation [2]. Treatment includes airway stabilization, oxygen, and hyperbaric therapy. Hyperbaric therapy ideally should be initiated as soon as possible.

We describe a case of a 46-year-old diving instructor who developed severe decompression illness following a long dive. As our facility did not have hyperbaric therapy available, the patient required transfer to another facility more than 100 miles away. Helicopter EMS (HEMS) transport was considered but not utilized given concerns for weather and possibly worsening decompression with the altitude change. The patient had demonstrated hemodynamic instability and relative hypoxemia, and the transporting ground EMS service was unable to perform rapid sequence intubation (RSI) if the patient's condition was to deteriorate, so the patient was intubated prior to transport. Within 24 hours he was found to have multiorgan failure and brain death despite hyperbaric therapy, no further hypoxia or hypovolemia, and no development of intracerebral gas. This case demonstrates the morbidity, mortality, and transport considerations of severe decompression illness.

## 2. Case Presentation

A 46-year-old male diving instructor was transported via EMS to a tertiary care ED with an initial chief complaint of shortness of breath after SCUBA diving for four hours in a freshwater spring. The patient was an experienced diver. He stated he had been diving at a depth of 30 meters (100 feet) for approximately four hours and described his ascent as “very conservative,” though further information regarding depth-time profile and breathing gas(es) was not reported. Several minutes after surfacing, the patient experienced shortness of breath and chest pain with associated nausea. When EMS arrived on scene, the patient was noted to have a blood pressure of 86/61, heart rate of 71, respiratory rate of 20, and pulse oximetry of 87% on room air. His hypoxemia improved to the mid-90s with administration of 15 liters/minute oxygen on a nonrebreather mask. He was then transported to a tertiary care center located approximately 30 miles (40 minutes driving) from the spring.

On ED presentation, the patient denied any medical history aside from several high blood pressure readings in the past; however he was not taking any medications. He was awake, oriented, and appropriately conversant. His vital signs were blood pressure of 123/69, pulse rate of 74, respiratory rate of 15, and pulse oximetry of 90% on the nonrebreather mask. He initially denied pain but did endorse generalized weakness and nausea and transient muscular pain.

**Figure 1:**
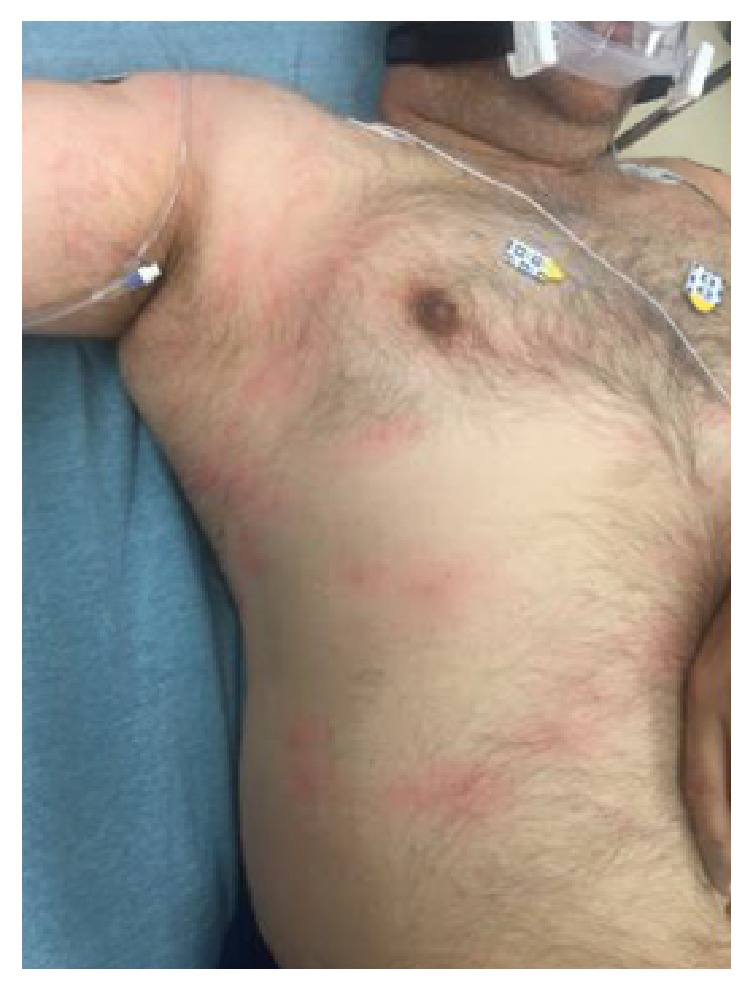
Cutis marmorata: skin mottling over chest and abdomen.

His physical exam was significant for mottling of the skin which progressed throughout his ED course (Figure 1). His breath sounds were equal and clear bilaterally. There was no tracheal deviation and breath sounds were clear. His abdomen remained soft and nontender. The patient's neurologic exam was nonfocal. Cranial nerves II through XII were intact, his speech was clear and appropriate, and he had symmetric normal grip strength and coordinated limb movement. He moved all four extremities with 5/5 strength and he had no ankle clonus. The patient's gait and truncal stability were not assessed. He did not experience pain upon range of motion of large joints.

**Figure 2:**
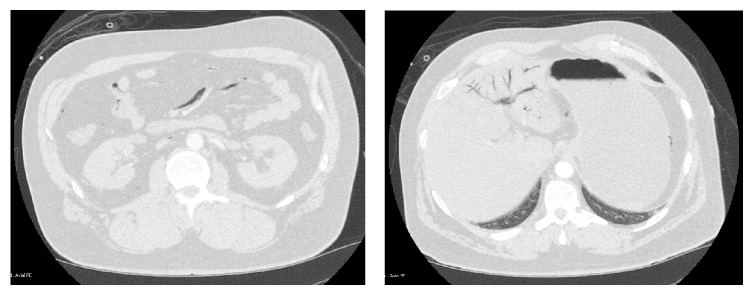
Mesenteric venous gas and pneumobilia.

**Figure 3:**
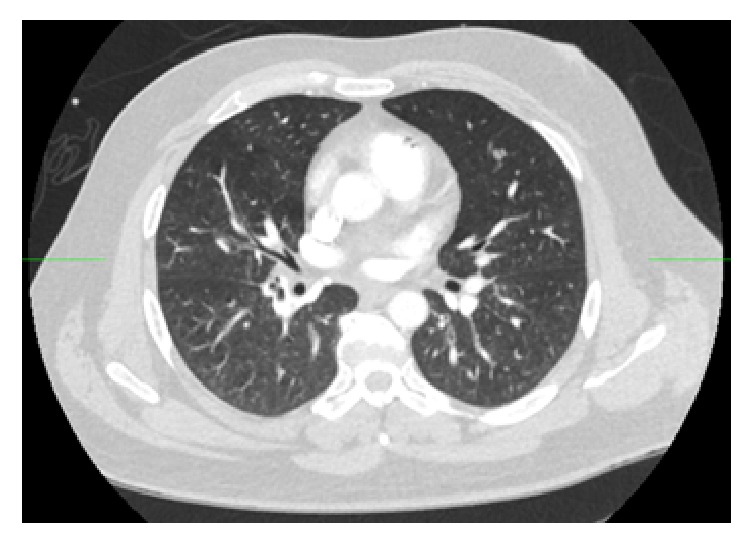
Gas in the main pulmonary artery and right lower lobe segmental pulmonary arteries.

While in the ED, the patient was changed from a nonrebreather mask to continuous positive airway pressure (CPAP) as a method of delivering a higher concentration of inhaled oxygen. Laboratory data included an elevated lactate of 2.25 mmol/L. A complete blood count revealed WBC of 23.9 × 10^9^ cells/L, hemoglobin of 17.7 g/dL, and a hematocrit of 55%. A basic metabolic panel was significant for creatinine of 1.54 mg/dl. A hepatic function panel showed mild elevations in AST and ALT at 55 U/L and 42 U/L, respectively. Coagulation studies were within normal limits. An arterial blood gas demonstrated pH 7.42, pCO2 35.7, pO2 65.1, bicarbonate 22.5, base deficit 0.8, and oxygen saturation 92.8. Carboxyhemoglobin and methemoglobin were within normal limits at 1.4 and 0.6, respectively. A chest X-ray was normal in appearance as was a computed tomography (CT) of the head. Decompression illness was suspected, but computed tomography angiography (CTA) was obtained for evaluation of other causes of hypotension and hypoxia. At approximately 2 hours after the diver surfaced, CTs revealed a large amount of air throughout the mesenteric and portal venous systems (Figure 2) with a moderate amount of air in the right lower lobe segmental pulmonary arteries (Figure 3). No air was seen in the pulmonary veins or the left heart. No intracerebral air was visualized on subsequent head CT.

Treatment consisted of administration of two liters of normal saline and fentanyl for transient limb pain and continuation of CPAP. There was no hyperbaric therapy available at our facility and the decision was made to transfer the patient to the nearest hyperbaric facility which was located 115 miles away. Transportation was arranged by way of ground EMS because weather conditions at that time precluded transportation by flight. There was also concern for possible worsening of the patient's developing decompression illness secondary to change in altitude if flown by helicopter. The EMS agency providing ground transportation did not have the capability to perform RSI should there be a change in medical condition; however they could transport patients already on a ventilator. Given the patient's hemodynamic instability and potential for rapid decompensation during a 2-hour transport, the decision was made to intubate the patient prior to transferring out of the facility. The potential risks and benefits were explained to the patient, and the patient gave informed consent to intubation and transport. He was intubated without difficulty and there were no hypoxic episodes during or after the procedure.

The patient remained hemodynamically stable without hypoxemia or hypotension while en route by ground EMS. Upon arrival to the receiving facility (7 hours after surfacing), the patient underwent immediate initiation of hyperbaric therapy. Sedation and analgesia were maintained so a formal neurologic examination was not performed prior to hyperbaric therapy initiation. In review of available records, it is unclear which treatment table was used. At approximately 12 hours after surfacing, sedation and analgesia were weaned for a formal neurological examination. Pupillary reflexes were found to be absent. The patient underwent repeat head CT (at approximately 14 hours after surfacing) which showed diffuse cerebral edema and sulci effacement in multiple vascular distributions. No intracerebral gas was observed. The patient was also noted to have undergone worsening renal function and shock liver. Consultation with neurology confirmed brain death and the patient's family subsequently withdrew care. The patient's profound neurologic injury was thought to be due to ongoing gaseous dissolution causing microvascular occlusion and widespread ischemia.

## 3. Discussion

Decompression illness is rare with the rate of occurrence estimated to be around 0.03% in recreational divers [3]. Decompression illness can present as a wide variety of manifestations and is divided into two types. Type I typically develops within an hour after surfacing and can present as generalized musculoskeletal pain, periarticular pain (“the bends”), a mottled and pruritic rash known as cutis marmorata, and even lymphedema [4]. Type II is characterized by neurological and cardiopulmonary symptoms [5]. Our patient presented with generalized fatigue, nausea, skin changes, and developed transient limb pain after a prolonged dive at approximately 100 feet. While being transported to our facility by EMS, the patient was also noted to have several transient hypotensive and hypoxic episodes. These manifestations were subtle; however the EMS response team maintained a high index of suspicion for decompression illness and began early resuscitative measures.

In situ bubble formation was extensive in this patient as demonstrated by multiple organ involvement seen on laboratory data (mild acute kidney injury, transaminitis, and hemoconcentration) and CTA imaging showing air within the mesenteric, portal venous system and pulmonary arteries. Given this widespread distribution, it is unlikely that a single embolic or iatrogenic source plus a structural cardiac abnormality (e.g., a patent foramen ovale) could account for all gas sites observed. Furthermore, noting that the second head CT (after hyperbaric therapy) did not show any further macroscopic intravascular gas formation, the patient's clinical decline and resultant cerebral ischemia were likely due to continued widespread microvascular occlusion.

The initial resuscitative step in a person with decompression illness should be the administration of oxygen, ideally at FiO2 of 100%. Pure oxygen serves to both improve tissue oxygenation and increase the partial pressure gradient favoring passage of nitrogen out of the bubbles formed during decompression [1]. It is reasonable for prehospital providers to consider noninvasive measures such as CPAP in patients not requiring intubation to achieve as high FiO2 as possible during transport. If noninvasive ventilatory support is utilized, providers should consider using minimal pressure settings (i.e., only enough to overcome device resistance) to reduce intrathoracic pressure which might increase left-to-right shunt and subsequently predispose to paradoxical embolism in patients with a patent foramen ovale. Furthermore, whether ventilation occurs via invasive or noninvasive means, reexpansion barotrauma may be a contributory factor in decompression illness, and thus practitioners should minimize peak pressures to any extent possible. Additionally, prehospital and emergency department providers should ensure adequate fluid resuscitation to ensure no compounding effects of dehydration or shock physiology [4].

The mainstay of treatment of decompression illness remains hyperbaric therapy. This should be initiated as soon as possible. The fastest available method of transportation is often by helicopter air ambulance. However, the manifestations of decompression illness may be exacerbated by decreases in atmospheric pressure [6]. Limited studies are available on establishing safe altitudes for patients with decompression illness, but current recommendations include ensuring that the cabin altitude does not exceed 500 feet (152 meters) above the departure location [7]. Unfortunately, this is the minimum helicopter altitude allowed under FAA Part 135 regulations for nighttime HEMS operations, and it is well below typical cruising altitude for long-distance interfacility transports. In lieu of clear evidence-based guidelines, treating medical teams must weigh the risks and benefits of transport via ground or air. Also, of note, HEMS pilot decision-making is independent of medical decision-making, so local weather conditions or daytime versus nighttime operations may preclude HEMS transport regardless of medical necessity. Finally, as delays in treatment may lead to worsening of symptoms, it is reasonable for initial prehospital providers to consider bypassing facilities that do not have hyperbaric treatment in favor of one that does. Medical directors with agencies likely to transport decompression patients might consider protocols for hospital bypass similar to those for trauma patients. For our patient, he might have benefited from an approximately 3-hour transport directly to a facility with available hyperbaric capability rather than the approximately 7-hour total time that included evaluation at our tertiary care facility. This difference may become more pronounced if transport from scene to final destination via HEMS is more clearly demonstrated as safe.

Clinical clues to recognition of decompression illness by EMS personnel involve historical and clinical factors. Because of the need for saturation of body tissue with nitrogen, deeper and longer dives make decompression illness more likely. These can be seen in recreational divers, technical divers, who may have used various mixed gases with depth, and occupational divers. Type 1 decompression illness, manifested as cutis marmorata or periarticular pain, may appear mild but serves as a marker that the diver needs evaluation for recompression therapy. The relief of joint pain when a sphygmomanometer cuff is inflated over the joint is suggestive of decompression illness, but the absence of pain relief with this maneuver does not exclude decompression illness. Type 2 decompression illness may manifest as paraparesis or paraplegia (“spinal bends”), vertigo (labyrinthine decompression illness), and pulmonary symptoms (“the chokes”) as well. Stroke-like symptoms may also develop from cerebral nitrogen bubble emboli in patients who have a paradoxical embolization via a patent foramen ovale or air embolization from pulmonary venous embolization after alveolar barotrauma [3].

## 4. Conclusion

Our case illustrates the importance of early recognition of the signs and symptoms of decompression illness. Oftentimes EMS responders are the first to encounter these patients and therefore must always maintain a high index of suspicion. 100% oxygen should be initiated in those with suspected decompression illness and prehospital personnel should also consider noninvasive measures such as CPAP if tolerated by the patient. Finally, these patients need to be transported to the nearest hyperbaric facility. Limited data exists on safe altitudes when transported by helicopter; however current studies show that flying at lower altitudes may be acceptable. Given the time-sensitive need for specialized resources, EMS services and medical directors should consider these regional risks when identifying those with possible decompression illness.
